# Exercise Stress Echocardiography in Athletes: Applications, Methodology, and Challenges

**DOI:** 10.3390/jcm12247678

**Published:** 2023-12-14

**Authors:** Stefano Palermi, Simona Sperlongano, Giulia Elena Mandoli, Maria Concetta Pastore, Matteo Lisi, Giovanni Benfari, Federica Ilardi, Alessandro Malagoli, Vincenzo Russo, Quirino Ciampi, Matteo Cameli, Antonello D’Andrea

**Affiliations:** 1 Public Health Department, University of Naples Federico II, 80131 Naples, Italy; stefano.palermi@unina.it; 2 Division of Cardiology, Department of Translational Medical Sciences, University of Campania Luigi Vanvitelli, 80131 Naples, Italy; sperlongano.simona@gmail.com (S.S.); vincenzo.russo@unicampania.it (V.R.); 3 Division of Cardiology, Department of Medical Biotechnologies, University of Siena, 53100 Siena, Italy; giulia.mandoli@unisi.it (G.E.M.); mariaconcetta.pastore@unisi.it (M.C.P.); matteo.lisi@unisi.it (M.L.); matteo.cameli@unisi.it (M.C.); 4 Section of Cardiology, Department of Medicine, University of Verona, 37126 Verona, Italy; giovanni.benfari@gmail.com; 5 Department of Advanced Biomedical Sciences, University of Naples Federico II, 80131 Naples, Italy; federica.ilardi@unina.it; 6 Division of Cardiology, Nephro-Cardiovascular Department, Baggiovara Hospital, University of Modena and Reggio Emilia, 41126 Modena, Italy; ale.malagoli@gmail.com; 7 Cardiology Division, Fatebenefratelli Hospital, 82100 Benevento, Italy; qciampi@gmail.com; 8 Department of Cardiology, Umberto I Hospital, 84014 Nocera Inferiore, Italy

**Keywords:** exercise stress echocardiography, sports cardiology, athletes, athlete’s heart

## Abstract

This comprehensive review explores the role of exercise stress echocardiography (ESE) in assessing cardiovascular health in athletes. Athletes often exhibit cardiovascular adaptations because of rigorous physical training, making the differentiation between physiological changes and potential pathological conditions challenging. ESE is a crucial diagnostic tool, offering detailed insights into an athlete’s cardiac function, reserve, and possible arrhythmias. This review highlights the methodology of ESE, emphasizing its significance in detecting exercise-induced anomalies and its application in distinguishing between athlete’s heart and other cardiovascular diseases. Recent advancements, such as LV global longitudinal strain (GLS) and myocardial work (MW), are introduced as innovative tools for the early detection of latent cardiac dysfunctions. However, the use of ESE also subsumes limitations and possible pitfalls, particularly in interpretation and potential false results, as explained in this article.

## 1. Introduction

**Figure 1:**
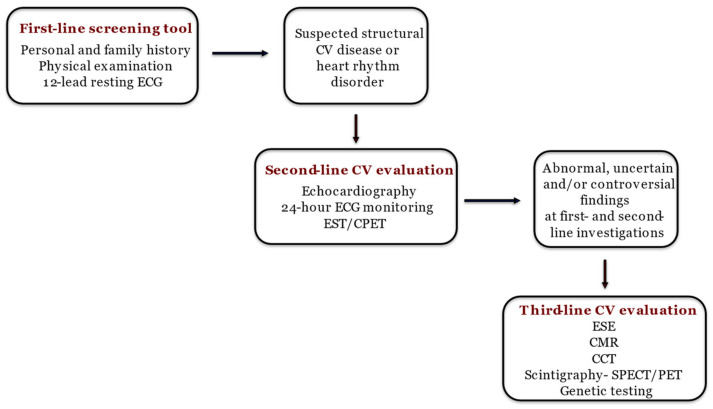
The step-by-step approach to diagnosing athlete’s heart [2]. ECG: electrocardiography; CV: cardiovascular; EST: exercise stress test; CPET: cardiopulmonary exercise test; ESE: exercise stress echocardiography; CMR: cardiac magnetic resonance; CCT: computed coronary tomography; SPECT: single photon emission computed tomography; PET: positron emission tomography.

Athletes put their cardiovascular systems under huge and repetitive effort, resulting in a heightened risk of developing cardiovascular diseases [1]. Additionally, their hearts undergo significant morphological, functional, and regulatory adaptations due to regular physical activity [1], leading to increased mass, cavity dimensions, and wall thickness with preserved systolic and diastolic function [2]. Despite preserved cardiac function, some of the mentioned physiological adaptations can overlap with pathological conditions, making a differential diagnosis quite challenging [3,4]. This similar phenotype, often referred to as the “grey zone”, requires a careful and precise diagnostic approach to ensure accurate identification and proper management, as proposed in Figure 1 [2].

When first- and second-line diagnostic evaluations yield unclear, abnormal, or debatable results, alternative cardiovascular (CV) diagnostic techniques can be instrumental in discriminating between normal adaptations and pathological conditions. However, owing to their significant expense and restricted accessibility, these methods are not commonly advised for routine use and should be employed based on specific clinical indications [2,5]. Stress imaging serves as a valuable method for revealing abnormal cardiac functional reserves or hidden pathologies that remain undetected during rest. This is particularly pertinent for athletes where there is a suspicion of arrhythmias and/or incipient cardiomyopathies [6]. Among stress imaging tests, exercise stress echocardiography (ESE) plays an important role in this diagnostic process. 

ESE is a reliable, safe, and noninvasive diagnostic tool that combines exercise stress tests with echocardiographic imaging to evaluate athletes’ CV responses during physical activity [7,8]. It has gained relevance in the evaluation of athletes, enabling the identification of CV abnormalities elicited only by exercise. It is advisable to perform ESE rather than pharmacological stress testing for any patient who can physically exercise, as it maintains the physiological CV response to exercise as well as the accuracy of the electrocardiogram (ECG) response and offers critical insights into functional status. Combining echocardiography with an exercise stress test facilitates the correlation of symptoms with CV stress and wall motion irregularities. The expanding evidence base advocating ESE’s utility beyond ischemia evaluation, its growing adoption in sports cardiology echocardiography labs, and its established diagnostic and prognostic significance underscore the necessity to develop specific guidelines for its application and execution [9]. 

In this review, we aim to explore the unique benefits and applications of ESE in the sports cardiology discipline, shedding light on its pivotal role in differentiating between athlete’s heart and other cardiovascular pathologies.

## 2. Indications for ESE in Athletes

Although there is ongoing debate in the literature about the optimal approach for mass preparticipation screening (PPS) [10], ESE is not recommended for this purpose. This is due to the rare occurrence of atherosclerotic coronary disease in young athletes and the limited diagnostic effectiveness of ESE in detecting anomalous coronary anatomy [4]. However, ESE is mainly indicated for the evaluation of a wide range of CV conditions in athletes since it gives information about cardiac function, reserve, exercise capacity, and arrhythmias [11] (Table 1). Indeed, wide availability, low cost, and the absence of radiation exposure make it the ideal third-line method [2,6]. The current European guidelines for sports cardiology identify ESE as an important test in cases where uninterpretable exercise stress results in the evaluation of coronary artery disease and to assess the severity and the hemodynamic response to exercise of a heart valve disease and possible complications of left ventricle (LV) hypertrabeculation [12]. A wide knowledge of athletes’ physiological echocardiographic features at rest is essential for discerning which individuals might benefit most from ESE. Echocardiographic adaptations to physical activity encompass several changes, such as a proportional enlargement of both left and right cardiac cavities, increased LV wall thickness and mass, and above-normal indices of both systolic and diastolic function [2].

### 2.1. Exercise-Induced Ischemia

A primary use of ESE is to identify exercise-induced ischemia in athletes experiencing chest pain or showing ECG anomalies. In such cases, it is crucial to rule out coronary artery disease (CAD) or congenital anomalies of the coronary arteries, both in terms of their origin and course [13]. For instance, CAD is diagnosed using ESE with moderate sensitivity and specificity (about 76 and 88%, respectively), comparing favorably with other stress-testing methods [14]. The choice between ESE and other third-line diagnostic modalities for the assessment of CAD in athletes is still a debated point in the literature (Table 2) [2].

### 2.2. Athlete’s Heart Grey Zone

In endurance athletes presenting with LV and/or right ventricular (RV) dilation and mildly reduced ejection fraction (EF) at rest, ESE can be employed to evaluate contractile reserve during exercise [3]. A marked increase in contractility with physical exertion (e.g., Δ LV EF > 5%) indicates physiological cardiac remodeling. In contrast, the absence or insufficient increase in contractility suggests a pathological condition (e.g., hypertrophic cardiomyopathy—HCM, dilated cardiomyopathy—DCM, LV noncompaction—LVNC, and arrhythmogenic cardiomyopathy—AC) (Table 3) [13,15]. A considerable EF increase suggests low EF at rest to be related to low preload and not to LV systolic dysfunction [11]. Abernethy et al. [16], involving 156 professional football players, demonstrated an increase in left ventricular ejection fraction (LV EF) during ESE across all subjects, regardless of their rest values. Tissue velocity imaging becomes a crucial tool when assessing cardiac function during exercise. Notably, elite rowers exhibited increased LV torsion but a decline in diastolic function and apical RV tissue Doppler-derived strain after high-intensity, short-duration exercises. In contrast, athletes engaged in mixed endurance and strength training showed improved diastolic function, as per tissue Doppler analysis, compared to non-athletes. Additionally, while weightlifters displayed a slight reduction in resting RV function measured by 2D and strain parameters, they showed significant improvement under stress conditions compared to inactive individuals. RV strain, measured using speckle-tracking echocardiography (STE), along with RV ejection fraction (fractional area change) and RV annular peak systolic velocity, demonstrate moderate to high accuracy in differentiating patients with AC from healthy adults [17,18]. During isometric exercise, highly trained resistance athletes have shown a greater increase in stroke volume and enhanced diastolic function compared to sedentary individuals, as reported in studies [19]. Millar et al. [20] explored the application of ESE in differentiating between athlete’s heart and DCM. Their findings revealed that during ESE, 96% of athletes in the grey zone demonstrated a rise in LV ejection fraction by more than 11% from baseline to peak exercise, in contrast to only 23% of DCM patients (*p* < 0.0001). Furthermore, a reduction in LV end-systolic volume during exercise was observed in both athletes and healthy subjects, but not in those with DCM or HCM. These results suggest that analyzing LV function during exercise could be a promising method for distinguishing between athlete’s heart and other pathological conditions [21]. 

In terms of systolic function, young endurance-trained athletes often exhibit a normal diastolic response during ESE. For instance, marathon runners display an increase in mitral E and e’ lateral upon exercising, which is linked to a modest rise in both E/e’ septal and E/e’ lateral while remaining within normal limits [22,23]. Elevated systolic pulmonary artery pressure (sPAP) may be induced by strenuous endurance exercise. Mirea et al. [24] found that 12.9% of the athletes they studied exhibited higher sPAP, which was further enhanced by bicycle ergometric stress effort and correlated with significant RV enlargement. Despite this, both conventional methods and STE indicated preserved RV function in these athletes. Indeed, a recent article [25] highlighted the utility of exercise stress echocardiography (ESE) in examining the pulmonary circulation and the right ventricle. This method has revealed prognostically important differences among healthy individuals, athletes, high-altitude dwellers, and patients with various cardiorespiratory conditions.

### 2.3. Hypertrophic Cardiomyopathy

**Figure 2:**
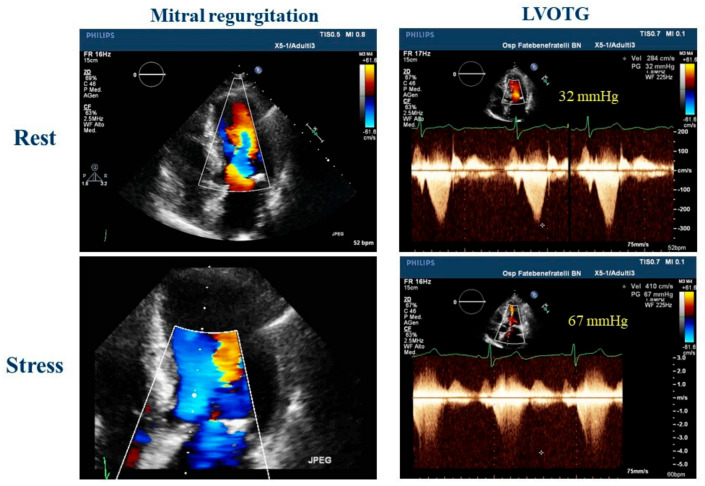
Exercise stress echocardiography in a 51-year-old master athlete, symptomatic for dyspnea during effort and with a family history of hypertrophic cardiomyopathy with a suspicious left ventricular hypertrophy; during effort, is it possible to observe an increase in mitral regurgitation and a significant left ventricle outflow tract obstruction that could explain the dyspnea. LVOTG: left ventricle outflow tract gradient.

ESE has been shown to have an important role in the diagnosis of HCM, an important cause of SCD in athletes [26]. In patients who do not exhibit outflow gradients at rest, ESE is the preferred method to induce obstruction. This technique not only has the potential to predict the future onset of progressive heart failure symptoms but also helps in distinguishing between patients with provocable obstruction and those without it. These distinctions have significant implications for guiding treatment options [27]. Furthermore, an LV outflow tract gradient exceeding 50 mmHg during or immediately after exercise in athletes with LV hypertrophy and symptoms like syncope or shortness of breath may indicate HCM [13,28]. Figure 2 shows the case of an athlete with LV hypertrophy and dyspnea. Indeed, Gaitonde et al. [29] showed how, compared with athletes, HCM patients had significantly higher LVOT peak gradients at rest and during ESE. Usually, there is no dynamic intraventricular obstruction with aerobic exercise in subjects with a structurally normal heart [30]. 

### 2.4. Valvular Heart Disease

The evaluation of athletes with valvular heart disease (VHD) deserves a special mention: in these cases, ESE may give complementary information on functional status and exercise tolerance, biventricular contractile reserve, and changes in hemodynamic and valvular functional parameters, including transvalvular gradients, regurgitation quantification, sPAP, and diastolic function [28]. Consequently, athletes with mild to moderate VHD should undergo ESE following a protocol that closely mirrors the level of physical exertion expected in their chosen sport [31] (Table 4).

### 2.5. Lung Screening

**Figure 3:**
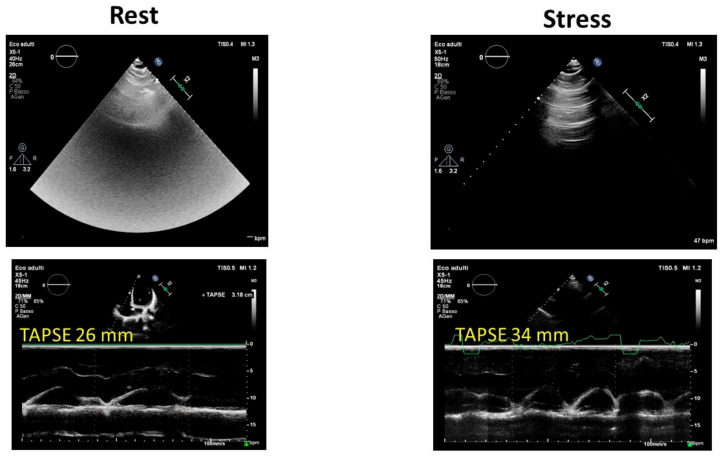
Exercise stress echocardiography in an endurance athlete: note the normal increase in tricuspid annular plane systolic excursion (TAPSE) and the mild increase in B-lines at peak effort. TAPSE: tricuspid annular plane systolic excursion.

B-lines assessed using lung ultrasound, commonly referred to as ultrasound lung comets, offer a straightforward and effective method to directly visualize extravascular lung water. A correct evaluation of the patient includes accurate scanning of the anterior and posterior chest and quantifying the number of B-line artifacts at each intercostal space. Stress lung ultrasound, which involves detecting B-lines during or immediately after exercise, is particularly valuable in two distinct scenarios: heart failure and extreme physiological conditions. In environments such as high-altitude trekking or among healthy elite apnea divers, scuba divers, underwater fishermen, and extreme athletes participating in triathlons or marathons, B-lines may be present even in the absence of pulmonary edema symptoms [32,33]. Therefore, during the ESE protocol for athletes, B lines should always be incorporated (Figure 3). Moreover, B-line evaluation during ESE was used to differentiate athletes and anabolic androgenic steroid users in a recent study by D’Andrea and colleagues [34], suggesting their potential use in the anti-doping evaluation of athletes.

## 3. Methodology of ESE

ESE should be preferentially performed as a supine bicycle exercise test or, in cases of unavailability, by using a standard treadmill or a cycle ergometer. Echocardiographic imaging must be performed at rest, during early and peak exercise, and recovery. As a general rule, patients who can engage in physical exercise should undergo testing with an exercise modality. This approach maintains the integrity of the electromechanical response and yields valuable insights into its functional status. Importantly, it accurately reflects an athlete’s physiological response to stress [28]. While ESE offers a dynamic assessment of cardiac function under physical stress, pharmacological stress tests may be preferred in athletes with limited exercise capacity or specific medical conditions [35]. Indeed, pharmacological stress is mostly used to exclude inducible myocardial ischemia (with dobutamine or dipyridamole), in the presence of a severe compromise of LV function, to determine myocardial viability (dobutamine), or when patients cannot exercise adequately [7]. An important advancement in cardiac imaging, especially relevant for athletes with suboptimal echocardiographic windows, is myocardial contrast stress echocardiography (MCSE). MCSE enhances the diagnostic clarity of ESE by improving endocardial border delineation, making it particularly valuable in cases where conventional ESE may be limited. The enhanced imaging provided by MCSE is particularly crucial in accurately assessing myocardial perfusion under stress conditions, offering a more detailed view of cardiac function [36].

During exercise, the athlete’s heart rate, blood pressure, and ECG have to be monitored continuously. 

The supine bicycle ergometer is often preferred over the treadmill or upright bicycle for exercise testing, particularly due to its compatibility with echocardiography during exercise. This approach is especially beneficial for athletes, who typically experience a rapid recovery phase with quick normalization of heart rate and blood pressure. Compared to upright bicycle or treadmill exercises, semisupine bicycle exercise is technically simpler, especially when assessing multiple stress parameters at peak exercise levels [28]. Its most significant advantage lies in its ability to capture images during each exercise phase rather than solely relying on post-exercise imaging. In the semisupine position, it becomes relatively straightforward to record images from multiple angles throughout various exercise intensities. Even with upright bicycle ergometer testing, acquiring apical images is feasible for most patients by having them lean forward over the handlebars or extend their arms [37]. Successful bicycle stress testing, however, depends on the patient’s cooperation in maintaining the correct cadence and coordinating the pedaling action. 

The stress protocol is typically designed to gradually increase the intensity of exercise, aiming at achieving a maximal level of exercise or a symptom-limited endpoint. The duration and intensity of exercise may be modified based on the athlete’s fitness level and the underlying medical conditions. Standard increases of 25 W every 2 min can be replaced by 50 W steps every 2 min in athletes to avoid a test duration over 12–15 min. Causes of test cessation may include intolerable symptoms, muscular exhaustion, high blood pressure (220/120 mmHg), symptomatic hypotension (>40 mmHg decrease), abnormal ventricular repolarization, and arrhythmias (supraventricular tachycardia, atrial fibrillation, frequent or complex ventricular arrhythmias) [28]. 

**Figure 4:**
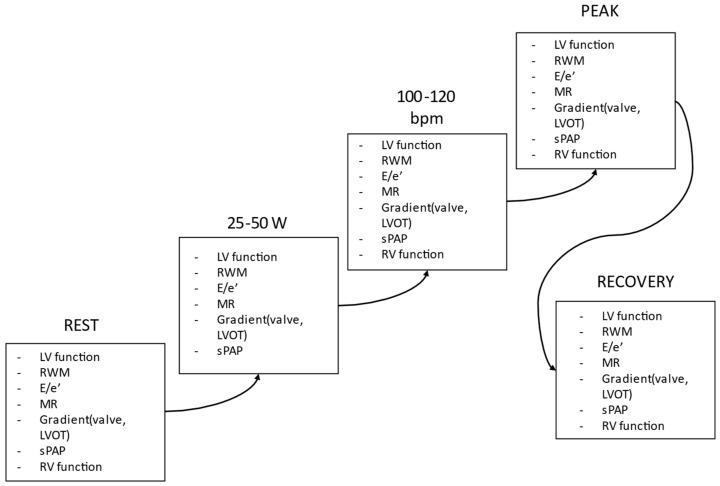
ESE protocol and parameters can be assessed at each stage [28]. Blood pressure, ECG recording, and clinical condition monitoring are continuously assessed. LV: left ventricle; LVOT: LV outflow tract; MR: mitral regurgitation; E/e’: ratio of early transmitral diastolic velocity to early TDI velocity of the mitral annulus; RWM: regional wall motion; RV: right ventricle; sPAP: systolic pulmonary artery pressure; W: watt; BPM: beats per minute.

**Figure 5:**
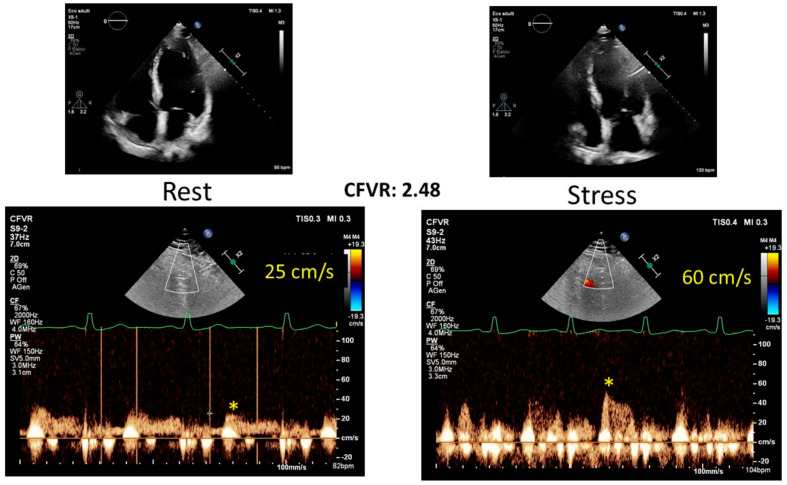
Exercise stress echocardiography in an endurance athlete; the global and regional left ventricular function was within normal limits at rest and during effort, with a normal increase in coronary flow and normal coronary flow reserve (>2). CFVR: coronary flow vascular reserve.

During ESE, various parameters can be evaluated, including biventricular function, transvalvular gradients and regurgitant flows, left and right heart hemodynamics encompassing SPAP, and ventricular volumes. Given the impracticality of assessing every possible parameter under stress, it is crucial to prioritize those most diagnostically relevant for each patient based on their importance [28] (Figure 4 and Table 5). For example, the chosen ESE protocol should be documented in the report. A typical response during both exercise and inotropic stress is characterized by an enhancement in function across all LV segments, coupled with an increase in LV EF and cardiac output [37]. The emergence or exacerbation of wall motion abnormalities in at least two consecutive LV segments signals ischemia, whereas an improvement by at least one grade in dysfunctional segments indicates recruitable viable myocardium. For patients without regional resting dysfunction, the global contractile reserve is usually defined by an increase of 5% or more in LV EF. Flow reserve, meanwhile, is characterized by a rise in forward stroke volume of 20% or more. It is essential to report any changes in cardiac function, such as variations in wall motion, EF, or global longitudinal function assessed by strain rate imaging, when available. Alterations in hemodynamic parameters like stroke volume, sPAP, E/e′, and LVOT gradients, as well as changes in the severity of valvular disease (including mitral regurgitation, aortic valve area, and pressure gradients), should be detailed based on the specific diagnostic question at hand. Additionally, blood pressure and heart rate data are critical to contextualizing contractile and hemodynamic responses. The assessment must always encompass the presence of viability and/or ischemia and the extent of coronary flow reserve. It is a useful diagnostic tool for detecting left anterior descending artery disease, and it is measured with pulsed doppler sampling of both the proximal and mid-distal tracts [28,38] (Figure 5).

The results of the ESE should be interpreted in the context of the athlete’s circumstances and risk factors. False positive and false negative results can occur, particularly in highly fit athletes, and the interpretation of ESE results requires expertise in both echocardiography and sports cardiology.

## 4. Emerging Techniques and Innovations in ESE

Over the past decade, LV global longitudinal strain (GLS)-assessed STE has gained recognition as a reliable tool for analyzing myocardial mechanics. It provides additional insights into cardiac performance beyond what traditional LV systolic function parameters, like EF, offer [39]. In endurance athletes, LV GLS tends to be lower at rest compared to healthy sedentary individuals, likely due to increased afterload, cardiac hypertrophy, and sinus bradycardia [40]. However, other studies have not found significant differences in GLS among athletes or have even reported higher GLS in athletes than in controls [41]. These variations in GLS may be attributed to several factors influencing strain measurements, such as preload, afterload, LV mass, sinus bradycardia, and the type of sport practiced. Gruca et al. [42] showed how GLS increased after a treadmill stress test in a cohort of 111 basketball athletes, and this could be potentially useful in the diagnostic process of athlete’s heart and other CV diseases. Indeed, several studies have shown that a reduction in LV GLS is uncommon in athlete’s heart, and that it cannot be regarded as a physiological adaptation to training and may be helpful to clarify the nature of cardiovascular adaptations in specific circumstances [43,44].

Recently, myocardial work (MW) has emerged as a novel noninvasive index for evaluating LV myocardial deformation through LV pressure–strain loop analysis. It represents an advancement over the GLS, offering deeper insights into LV performance under varying effort levels by incorporating afterload and measuring myocardial efficiency [45]. MW provides additional information beyond EF and strain under different LV-loading conditions, such as in athletes. Analyzing MW in endurance athletes is particularly useful for assessing LV myocardial deformation and contractile reserve in a manner less dependent on loading [46].

Furthermore, integrating stress echocardiography with GLS and MW analysis shows promise for the early detection of subclinical cardiac dysfunction not evident at rest [47]. This approach has broad applications in diagnosing athlete’s heart. Isometric effort enhances strain, particularly in the mid-apical LV segments, indicating a greater regional function reserve [48]. As suggested by the recent study of D’Andrea et al. [49], a comprehensive strain study, both at rest and during ESE, could play an incremental role in aiding clinicians in managing and making decisions for the endurance of the athletes’ hearts. Additionally, the mechanical dispersion of GLS, measured as the standard deviation of the time to peak longitudinal strain across all LV segments, appears to be a promising diagnostic tool for HCM in athletes [50].

Recent studies also explore the potential of combining ESE with other techniques, such as cardiopulmonary exercise testing (CPET) [51] and the latest application of artificial intelligence (AI)-related ESE [52,53]. In a recent study, Upton et al. [54] demonstrated the significant role of AI-based methods in enhancing the accuracy, confidence, and reproducibility of stress echocardiography interpretations.

## 5. Limitations of ESE in Athletes

Although ESE is a useful diagnostic tool for athletes, some limitations should be mentioned. ESE requires specialized equipment and expertise and may not be widely available or affordable in all settings. ESE also requires a significant time commitment, as it typically involves a lengthy imaging protocol and monitoring of the athlete.

One of the major limitations of ESE in athletes is the potential for false positive results. Exercise-induced changes in LV function, such as wall motion abnormalities, can occur in healthy athletes, and these changes can be misinterpreted as evidence of underlying cardiovascular disease. This can lead to unnecessary testing, treatment, and restrictions on athletic participation.

Another limitation of ESE in athletes is the potential for false negative results. ESE may fail to detect CV disease in athletes, particularly in those with early or mild forms of the disease. This can lead to missed diagnoses, delayed treatment, and an increased risk of complications.

Interpretation of ESE results in athletes can be challenging, particularly in those with high levels of physical fitness. Athletes may have LV hypertrophy, LV cavity enlargement, or LV wall motion abnormalities that are unrelated to underlying cardiovascular disease. Distinguishing between normal physiological adaptations and pathological changes can be difficult and may require additional testing, such as cardiac magnetic resonance imaging (MRI).

## 6. Conclusions

ESE plays a vital role in the cardiovascular evaluation of athletes. Combining exercise stress testing with echocardiographic imaging, provides valuable information regarding cardiac function, reserve, exercise capacity, and arrhythmias. ESE is a reliable, safe, and noninvasive diagnostic tool that can unmask covert pathological features that may not be evident at rest. Its increasing implementation in sports cardiology’s echocardiography laboratories speaks to its recognized diagnostic and prognostic value. While further research is needed to establish comprehensive guidelines for its application and performance, ESE’s unique ability to differentiate between athlete’s heart and other cardiovascular pathologies makes it an indispensable component of the diagnostic process.

## Figures and Tables

**Table 1:** Main indications of ESE in athletes.

Detection of exercise-induced ischemia
Differential diagnosis in grey zones: LV wall thickening, LV/RV dilatation, LV hypertrabeculation
Diagnosis and prognosis of HCM
Diagnosis and prognosis of heart valve diseases
Lung B line screening

LV: left ventricle; RV: right ventricle; HCM: hypertrophic cardiomyopathy.

**Table 2:** Details of third-line diagnostic modalities for the diagnosis of CAD in athletes [2].

	Pros	Cons
ESE	- Physiological activation of the CV system - Non-radiation imaging modalities - Low cost	- Requires specific and expensive equipment - Motion artifacts - Limited skeletal muscle fatigue in individuals accustomed to cycling
CCT	- High spatial resolution - High-quality multiplanar reconstruction in any orientation - Short examination time	- Costs - Limited access - Radiation dose - Low temporal resolution
Nuclear imaging	- Excellence accuracy	- Costs - Radiation dose - Low specificity in competitive athletes

ESE: exercise stress echocardiography; CV: cardiovascular; CCT: cardiac computed tomography.

**Table 3:** The use of ESE in the differential diagnosis of grey zones in athlete’s heart: LV wall thickening, LV/RV dilatation, LV hypertrabeculation [2].

Parameters during Effort	Findings Suggestive of Normal Heart	Finding Suggestive of CV Pathologies
Contractile reserve	Significant improvement	Absent or subnormal improvement
Dynamic obstruction	No dynamic intraventricular obstruction	LVOTO or mid-cavity obstruction
Diastolic function	Normal/supranormal diastolic function indexes	Diastolic dysfunction
Heart valve diseases	Absent	Dynamic/functional new onset/worsening valve diseases
Ischemia	Absent	Inducible ischemia
Lung echocardiography	Normal	Pulmonary congestion

ESE: exercise stress echocardiography; LV: left ventricle; LVOTO: left ventricle output tract obstruction; RV: right ventricle.

**Table 4:** The use of ESE in the evaluation of athletes with valvular heart disease [31].

	MVP	MR	MS	TR/TS
ESE parameters to be evaluated	Evaluation of sPAP increase during exercise	Evaluation of hemodynamic consequences and arrhythmias during exercise	Symptoms, sPAP, and MV dynamic gradient evaluation during exercise	Symptoms, sPAP, and TV dynamic gradient evaluation during exercise

MR: mitral regurgitation; MS: mitral stenosis; MV: mitral valve; MVP: mitral valve prolapse; sPAP: systolic pulmonary artery pressure; TR: tricuspid regurgitation; TS: tricuspid stenosis; TV: tricuspid valve; ESE: exercise stress test.

**Table 5:** Targeted parameters to be assessed during ESE [28].

Indications	Query	Parameters to Be Acquired	Levels of Image Acquisition	Possible Results	Report
HCM	LVOTO diastolic dysfunction dynamic MR inducible ischemia as a reason for symptoms, or to plan treatment lifestyle advice	CW Doppler LVOT velocity TR CW Doppler for SPAP PW Doppler (E and A) PW Tissue Doppler (e′) color flow Doppler for MR LV view RWMA	Baseline low workload peak exercise immediately after exercise	LVOTO +sPAP increase E/e′ increase +sPAP increase MR appearance/increase RWMA	Exertion-induced LVOTO Diastolic dysfunction Dynamic MR Inducible ischemia
DCM	Contractile reserve inducible ischemia diastolic reserve SPAP change dynamic MR pulmonary congestion	LV views PW Doppler (E and A) PW tissue Doppler (e′) TR CW Doppler for SPAP Color flow Doppler for MR lung images	Baseline low workload peak exercise	Contractility increase No contractility increase E/e′ increase +sPAP increase RWMA Lung comets MR increase/decrease	Contractile reserve No contractile reserve Pulmonary congestion Dynamic MR functional MR Inotropic reserve No inotropic reserve
Primary mitral regurgitation	Nonsevere MR with symptoms	Color flow Doppler for MR LV views TR CW Doppler for SPAP	Baseline low workload peak exercise	MR increase No MR increase	Severe MR with symptoms Symptoms unrelated to MR
Aortic regurgitation	Nonsevere AR with symptoms	LV views Color flow Doppler for MR TR CW Doppler for SPAP	Baseline low workload peak exercise	RWMA sPAP increase MR appearance/increase	Inducible ischemia pulmonary hypertension dynamic MR
Symptomatic athlete	Assess response to exercise and symptoms	LV views LVOT CW Doppler for LVOTO TR CW Doppler for SPAP Color flow Doppler for MR lung images	Baseline low workload peak exercise	RWMA LVOTO Pathologic sPAP increase MR appearance/increase Lung comets	Induced ischemia LVOTO Pulmonary hypertension Dynamic MR Pulmonary congestion

AR: aortic regurgitation; AV: aortic valve; CW: continuous wave; DCM: dilated cardiomyopathy; EF: ejection fraction; HCM: hypertrophic cardiomyopathy; LV: left ventricle; LVOT: left ventricle output tract; LVOTO: LV outflow tract obstruction; MR: mitral regurgitation; MV: mitral valve; PW: pulse wave; RV: right ventricle; RWMA: regional wall motion abnormality; sPAP: systolic pulmonary artery pressure; TAPSE: tricuspid annular systolic plane excursion; TR: tricuspid regurgitation.

## Data Availability

Data are available, on reasonable request, with request to the corresponding author.
